# Identification of Differentially Expressed Genes and miRNAs Associated with Esophageal Squamous Cell Carcinoma by Integrated Analysis of Microarray Data

**DOI:** 10.1155/2020/1980921

**Published:** 2020-07-01

**Authors:** Lemeng Zhang, Jianhua Chen, Tianli Cheng, Hua Yang, Changqie Pan, Haitao Li

**Affiliations:** Thoracic Medicine Department 1, Hunan Cancer Hospital, Changsha, Hunan Province, China

## Abstract

To identify candidate key genes and miRNAs associated with esophageal squamous cell carcinoma (ESCC) development and prognosis, the gene expression profiles and miRNA microarray data including GSE20347, GSE38129, GSE23400, and GSE55856 were downloaded from the Gene Expression Omnibus (GEO) database. Clinical and survival data were retrieved from The Cancer Genome Atlas (TCGA). Kyoto Encyclopedia of Genes and Genomes (KEGG) pathway enrichment analysis of differentially expressed genes (DEGs) was analyzed via DAVID, while the DEG-associated protein-protein interaction network (PPI) was constructed using the STRING database. Additionally, the miRNA target gene regulatory network and miRNA coregulatory network were constructed, using the Cytoscape software. Survival analysis and prognostic model construction were performed via the survival (version 2.42-6) and rbsurv R packages, respectively. The results showed a total of 2575, 2111, and 1205 DEGs, and 226 differentially expressed miRNAs (DEMs) were identified. Pathway enrichment analyses revealed that DEGs were mainly enriched in 36 pathways, such as the proteasome, p53, and beta-alanine metabolism pathways. Furthermore, 448 nodes and 1144 interactions were identified in the PPI network, with *MYC* having the highest random walk score. In addition, 7 DEMs in the microarray data, including miR-196a, miR-21, miR-205, miR-194, miR-103, miR-223, and miR-375, were found in the regulatory network. Moreover, several reported disease-related miRNAs, including miR-198a, miR-103, miR-223, miR-21, miR-194, and miR-375, were found to have common target genes with other DEMs. Survival analysis revealed that 85 DEMs were related to prognosis, among which hsa-miR-1248, hsa-miR-1291, hsa-miR-421, and hsa-miR-7-5p were used for a prognostic survival model. Taken together, this study revealed the important roles of DEGs and DEMs in ESCC development, as well as DEMs in the prognosis of ESCC. This will provide potential therapeutic targets and prognostic predictors for ESCC.

## 1. Introduction

Esophageal carcinoma (EC) remains the sixth leading cause of cancer-associated mortality worldwide, with approximately 442,000 new cases and 440,000 mortalities globally in 2014 [1, 2]. As the predominant subtype of EC, esophageal squamous cell carcinoma (ESCC) is frequently diagnosed in Eastern Asian countries, including China, where it accounts for 95% of all EC cases [3, 4]. A series of risk factors, such as tobacco smoking and consumption of alcohol and salted vegetables, are reported to be associated with the high incidence of ESCC [5]. Currently, despite advances in diagnosis and treatment of ESCC, its prognosis remains poor, with a 5-year overall survival rate of less than 40% [6, 7]. Therefore, identification of the molecular mechanisms underlying the progression and prognosis of ESCC is of utmost importance.

As a gene detection technique, gene profiling or gene chips have been widely used during the last decade for the comprehensive screening of differentially expressed genes (DEGs) [8]. With the development and application of gene chips, more and more data have been generated and stored in public databases, which will provide valuable clues for new research. Currently, an increasing number of studies have reported the identification of DEGs in ESCC. For example, Yuan et al. [9] analyzed the gene expression profile in the GSE20347 dataset, identifying a total of 538 DEGs. Likewise, Xing and Liu [10] identified 1204 DEGs based on the GSE23400 dataset generated from ESCC and matched mucosa tissues. Furthermore, Hu et al. [11] focus on biallelic loss and its relation to mRNA expression based on the GSE38129 array data. Recently, differentially expressed miRNAs (DEMs) has been reported to be associated with differentiation, invasion, and metastasis of patients with ESCC [12]. Moreover, miRNA expression profiling analysis was also recently performed [13]. Jang et al. [14] identified prognostic markers for postoperative recurrence of ESCC by building an easy-to-use prognostic model with three small noncoding RNAs (sncRNAs) based on the GSE55856 dataset. However, the majority of these reports were based on a single dataset, which leads to the results being limited or inconsistent. Hence, the combination of bioinformatics methods and expression profiling techniques using different datasets may provide valuable information for the development of therapeutic strategies for patients with this disease.

In the present study, we obtained the original microarray data of the GSE20347, GSE38129, and GSE23400 datasets, as well as the miRNA microarray data of the GSE55856 dataset from the Gene Expression Omnibus (GEO). Clinical data and survival data were retrieved from The Cancer Genome Atlas (TCGA). Subsequently, the DEGs or DEMs were screened. Pathway enrichment analyses were performed, and protein-protein interaction (PPI) networks were created for the DEGs, in order to identify key genes and their biological function in ESCC. Additionally, the miRNA target gene regulatory network and miRNA coregulatory network were constructed to investigate the underlying functions of these miRNAs. As such, based on survival analysis of DEMs and univariate Cox analysis, a prognostic survival model based on the expression of different miRNAs was constructed.

## 2. Materials and Method

### 2.1. Microarray Data Collection and Preprocessing

Gene expression profiles from the GSE20347 (34 samples), GSE38129 (60 samples), and GSE23400 (106 samples) datasets between ESCC samples and matched normal controls were obtained from the GEO (http://www.ncbi.nlm.nih.gov/geo/) database using the Affymetrix Human Genome U133A 2.0 Array platform. In addition, the miRNA microarray GSE55856 dataset, which is composed of 216 samples (108 ESCC samples and 108 normal controls), was obtained using the Affymetrix Multispecies miRNA-2_0 Array.

For the preprocessing of the gene expression profile chip, the raw data of the GSE20347, GSE38129, and GSE23400 datasets were preprocessed using the R Affy package (version: 1.46.1) with a standardized RMA method [15]. The processing included background corrections, normalization, and calculation of gene expression. Afterwards, the probe ID was converted to a gene symbol, with probes that had no corresponding gene symbols being removed. As for the case where multiple probes correspond to the same gene symbol, we selected the mean value of the probes as the final gene expression value. The preprocessing of miRNA microarray data was done in a similar manner using the miRNA chip platform of Affy.

### 2.2. Identification of DEGs and DEMs

The limma package of R (version: 3.30.2) [16] was used to identify genes or miRNAs that were significantly differentially expressed between the tumor and normal tissues. *P* < 0.05 and log2 fold change (FC) ≥ 0.58 were selected as the cutoff values for statistically significant DEGs or miRNAs. Subsequently, we selected 3 groups of DEGs and then analyzed whether the genes were also significantly differentially expressed in the 3 datasets.

### 2.3. Prediction of DEGs Based on a Meta-analysis

By screening DEGs based on a meta-analysis, more reliable DEGs can be obtained due to the collection of multiple experimental datasets and enhancement of statistical ability. In order to integrate the DEGs that were combined in the three datasets, the MetaDE package of R (version: 1.0.5) was used [17]. Gene expression values were examined for heterogeneity with statistic parameters including tau^2^, *Q* value, and *QP* value. Criteria standards of tau^2^ = 0 and QPval > 0.05 were selected as the homogeneity test parameter. A *P* value of < 0.05 was the threshold for a significant difference in gene expression. Moreover, a heatmap was generated with the pheatmap [18] R package (version: 3.25).

### 2.4. Pathway Enrichment Analysis for DEGs

To investigate the biofunctions of DEGs, Kyoto Encyclopedia of Genes and Genomes (KEGG) enrichment analysis was performed using DAVID (version: 6.8) [19]. *P* < 0.05 was selected as the cutoff criterion.

### 2.5. Identification of PPI Network and Key Genes

To better understand the interactions of the DEGs, the Search Tool for the Retrieval of Interacting Genes (STRING) database (version: 10) was employed to develop a DEG-encoded PPI [20], with a reliability threshold of >0.9. The prediction methods were derived from the neighborhood, gene fusion, cooccurrence, coexpression, experiments, databases, and text mining. The Cytoscape software (version: 3.2.1) was utilized to construct the PPI.

Next, the random walk algorithm was used to analyze important nodes in the PPI network. Briefly, the random walk was started at the seed node and the importance of each node was expressed by calculating the frequency of each node after the random walk between nodes in the network. The corresponding higher frequency genes may be candidate genes that have important physiological regulatory functions. The RWOAG package of R [21] was used to calculate the network node score.

### 2.6. Construction of the miRNA-Target Gene Regulatory Network

Mirwalk2 (http://zmf.umm.uni-heidelberg.de/apps/zmf/mirwalk2) [22] was used for the prediction of target genes regulated by miRNAs, and differentially expressed target genes were filtered by using the “validated target” module. Based on the data of differentially expressed miRNAs and DEGs, the opposite relationship pairs (upregulated miRNA-downregulated gene or downregulated miRNA-upregulated gene) were selected from the miRNA-target gene data. The regulatory network of miRNA-target genes was constructed using the Cytoscape software [23]. Meanwhile, we screened several miRNAs from the miR2Disease database (http://www.mir2disease.org/) [24] which were reported to be related to ESCC.

### 2.7. Functional Analysis of miRNAs

In order to obtain information regarding the pathways associated with the identified miRNAs, we performed KEGG pathway analysis for differentially expressed target genes using the clusterProfiler package of R (version: 3.3.1). The enrichment significance *P* value was corrected with the BH method and a *P* value of less than 0.05 was considered to be significant.

### 2.8. Construction of miRNA Coregulatory Network

Based on the regulatory network of miRNAs and their target genes, miRNA pairs that regulate the same target genes were screened to construct the coregulatory network between miRNAs.

### 2.9. Survival Analysis of DEMs

Clinical and survival data from 251 patients with ESCC were retrieved from TCGA, which were downloaded from the database of University of California Santa Cruz (UCSC) Xena (https://xenabrowser.net). Moreover, miRNA-seq data was downloaded from http://gdac.broadinstitute.org/runs/stddata__2016_01_28/data/ESCA/20160128/.

In general, TCGA data directly downloaded cannot be directly analyzed. Therefore, in order to link different data, we need to match, select, and delete different data by screening samples. In this study, the DEMs obtained from the integration analysis of different GEO databases intersected with the miRNA-seq data filenames downloaded by TCGA using an R package [25]. Additionally, miRNAs with value = 0 in more than half of the total samples were removed from the existing intersection data.

The optimal miRNA cutoff was determined via the surv_cutpoint of survminer (version 0.4.3) of R package, with >optimal cutoff being considered high expression and <optimal cutoff being considered low expression. Survival analysis was conducted with the survival (version 2.42-6) R package, and *P* values < 0.05 were taken as the threshold. miRNAs with significant correlation to prognosis were selected and survival curves were made.

### 2.10. Univariate Cox Analysis and Prognostic Model Construction

Univariate survival Cox analysis was continued for miRNAs significantly correlated with survival, and miRNAs with *P* values < 0.05 were used for the construction of the prognostic model.

After univariate analysis, there were still many significant univariate factors, which were not conducive to inclusion in the prognostic model. Therefore, some dimensionality reduction methods were adopted to select the most important univariate factors to be included in the prognostic model for downstream analysis. In this study, the rbsurv R package was used to investigate the robustness of univariate survival analysis. Briefly, 3/4 samples were randomly selected as training data and the remaining 1/4 samples as validation data. Multivariate Cox analysis was carried out on the obtained models in the test training set and verification set, and risk scores for survival verification of the model were obtained. Finally, the overall evaluation effect of the model on prognosis was checked.

## 3. Results

### 3.1. Identification of DEGs and DEMs

After data preprocessing, a total of 2575, 2111, and 1205 DEGs between ESCC and normal tissues were identified in the gene expression profile of the GSE20347, GSE38129, and GSE23400 datasets, respectively, based on the cutoff criteria. Moreover, 226 DEMs were identified in GSE55856, of which 190 were upregulated and 36 were downregulated.

### 3.2. Meta-analysis of DEGs and Hierarchical Clustering

**Figure 1:**
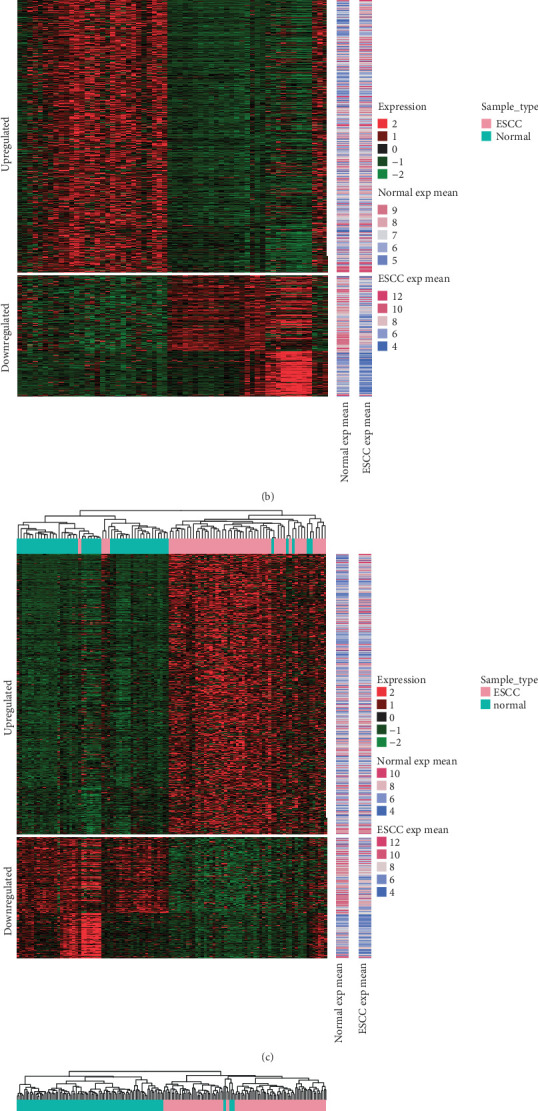
Heatmap clustering of the differentially expressed genes (DEGs) and miRNAs (DEMs) between ESCC and normal tissues samples in the GSE20347 (a), GSE38129 (b), GSE23400 (c), and GSE55856 (d) datasets. “Red” represents high relative expression and “green” represents a low relative expression.

Based on the meta-analysis, 1001 DEGs, including 700 upregulated genes and 301 downregulated genes were obtained. As shown in Figure 1, hierarchical clustering revealed that the DEGs obtained from the meta-analysis and DEMs could effectively cluster the samples from the GSE20347, GSE38129, GSE23400, and GSE55856 datasets, which suggests that the ESCC samples could easily be distinguished from the normal controls by analyzing the DEGs or miRNAs.

### 3.3. Functional Enrichment Analysis of DEGs Screened from the Meta-analysis

**Figure 2:**
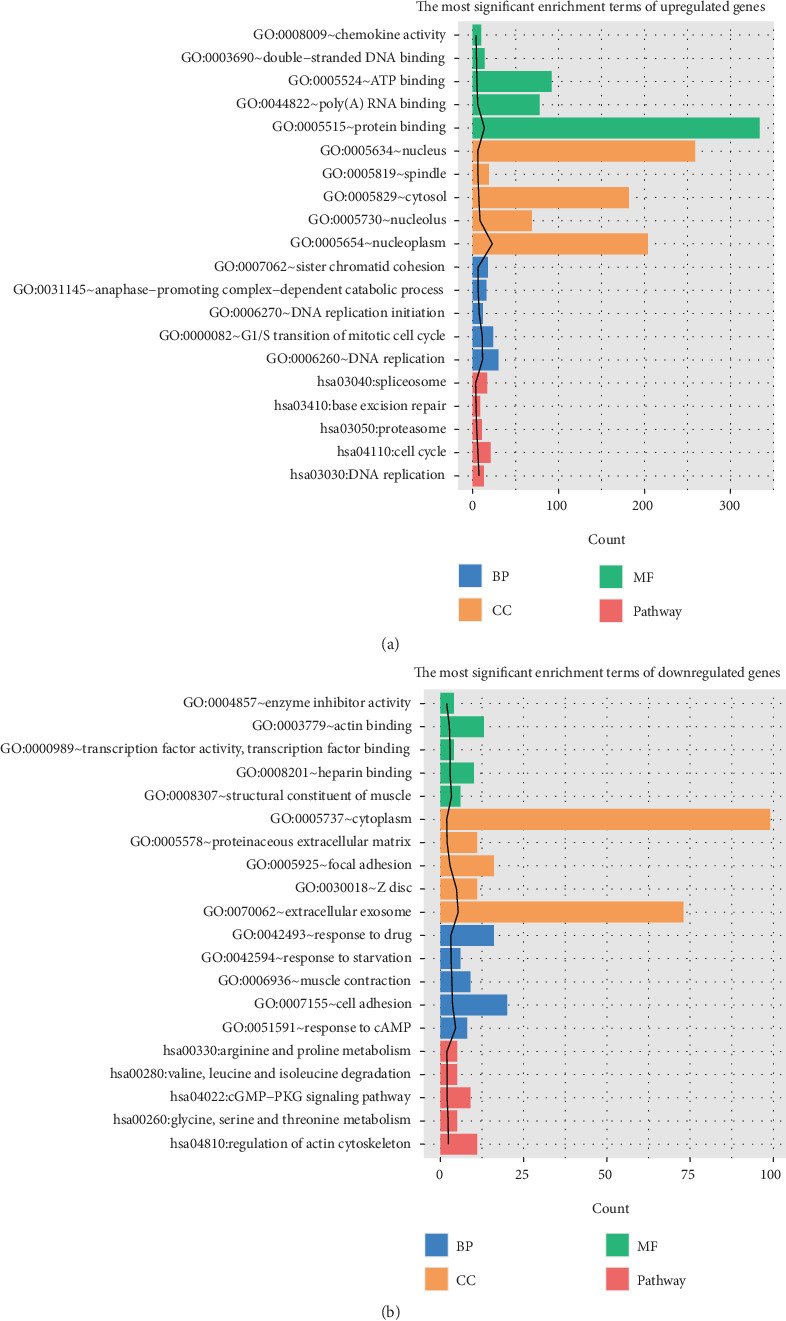
DEG functional enrichment analysis in ESCC. Upregulated (a) and downregulated (b) DEGs were enriched in four functional categories, including pathways, biological processes, cellular components, and molecular functions.

As illustrated in Figure 2, DEGs were classified into four functional categories, including pathways, biological process, cellular components, and molecular function. KEGG pathway analysis revealed that the upregulated DEGs were mainly enriched in 18 pathways, including DNA replication, cell cycle, proteasome, base excision repair, and the spliceosome signaling pathway (all, *P* < 0.05; Table 1). Similarly, 18 KEGG pathways were significantly enriched in downregulated DEGs, such as regulation of actin cytoskeleton, glycine, serine, and threonine metabolism, cGMP-PKG signaling pathway, valine, leucine, and isoleucine degradation and the arginine and proline metabolism signaling pathway (all, *P* < 0.05).

### 3.4. Identification of Key Candidate Genes and Pathways by DEGs PPI Network Analysis

**Figure 3:**
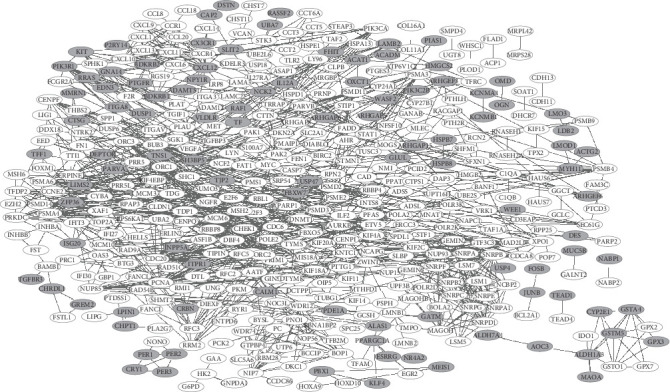
DEGs protein-protein interaction (PPI) network in ESCC. There were 448 nodes and 1144 interactions identified in the network. White represents upregulated genes and gray represents downregulated genes.

As shown in Figure 3, a series of DEGs were filtered into the PPI network, which contained 448 nodes and 1144 interaction pairs. Among the nodes, the key candidate node genes were identified by filtering the random walk score. The top 20 nodes, including 17 upregulated and 3 downregulated genes were summarized in Table 2. Among these DEGs, the MYC protooncogene (*MYC*) had the highest score.

### 3.5. Regulatory Network of miRNA Target Genes

**Figure 4:**
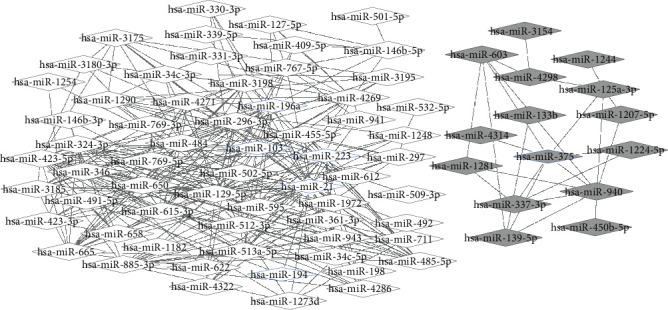
MiRNA-target gene regulatory network. There were 72 upregulated miRNAs and 130 downregulated target genes, as well as 19 downregulated miRNAs and 133 upregulated target genes in the network. Gray represents a downregulated expression, while white represents an upregulated expression. Ovals represent the differentially expressed target gene, and diamonds represents the DEMs (blue edge represents miRNAs reported to be disease related).

According to the screening principles of an upregulated miRNA-downregulated gene or downregulated miRNA-upregulated gene, we constructed the miRNA-target gene regulatory network. As shown in Figure 4, a total of 72 upregulated miRNAs which targeted 130 downregulated genes, as well as 19 downregulated miRNAs which targeted 133 upregulated genes were filtered in the network. Based on the miRNAs associated with ESCC obtained from miR2Disease (http://www.mir2disease.org/), 8 miRNAs, including miR-196a, miR-21, miR-205, miR-194, miR-103, miR-223, miR-203, and miR-375, were found to be significantly differentially expressed in miRNA microarray datasets. Among these miRNAs, 7 miRNAs excluding miR-203 were found in the network. Moreover, the gene with the highest score in the PPI network, *MYC*, was coregulated by miR-125a-3p, miR-940, and miR-375, among which miR-375 has been reported to be related to ESCC.

### 3.6. Coregulatory Network between miRNAs

**Figure 5:**
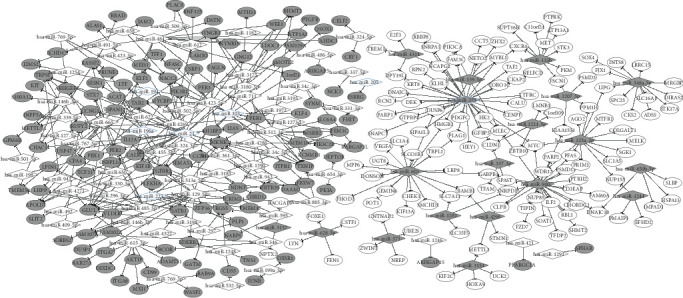
The coregulatory network between miRNAs. Several reported disease-related miRNAs shared common target genes with other DEMs. Gray represents a downregulated expression and white represents an upregulated expression. The blue thickened edge represents miRNAs reported to be disease related.

In order to construct the coregulatory network, the miRNAs that regulated the same target gene were identified via the miRNA-target gene regulatory network. As illustrated in Figure 5, several miRNAs which are reported to be disease related, such as hsa-miR-198a, hsa-miR-103, hsa-miR-223, hsa-miR-21, hsa-miR-194, and hsa-miR-375, had common target genes with other DEMs. These miRNAs have played an essential in proliferation, invasion, and metastasis of malignant disease, which is closely related to pathogenesis and prognosis.

### 3.7. Survival Analysis of DEMs

After processing of the TCGA data described above, a total of 184 cancer samples and 174 differentially matched miRNAs were obtained. Survival analysis revealed that there were 91 DEMs significantly correlated with the outcome of ESCC patients (Supplementary Table 1).

### 3.8. Univariate Cox Analysis and Prognostic Model Construction

A total of 13 DEMs were obtained after the univariate Cox analysis. According to the model analysis, a prognostic survival model with 4 DEMs, including hsa-miR-1248, hsa-miR-1291, hsa-miR-421, and hsa-miR-7-5p was obtained. Among these miRNAs, hsa-mir-1248, hsa-miR-1291, and hsa-miR-421 were the DEMs in the GEO data. Moreover, hsa-miR-7-5p was concentrated as the precursor of hsa-miR-7 in the GEO data.

**Figure 6:**
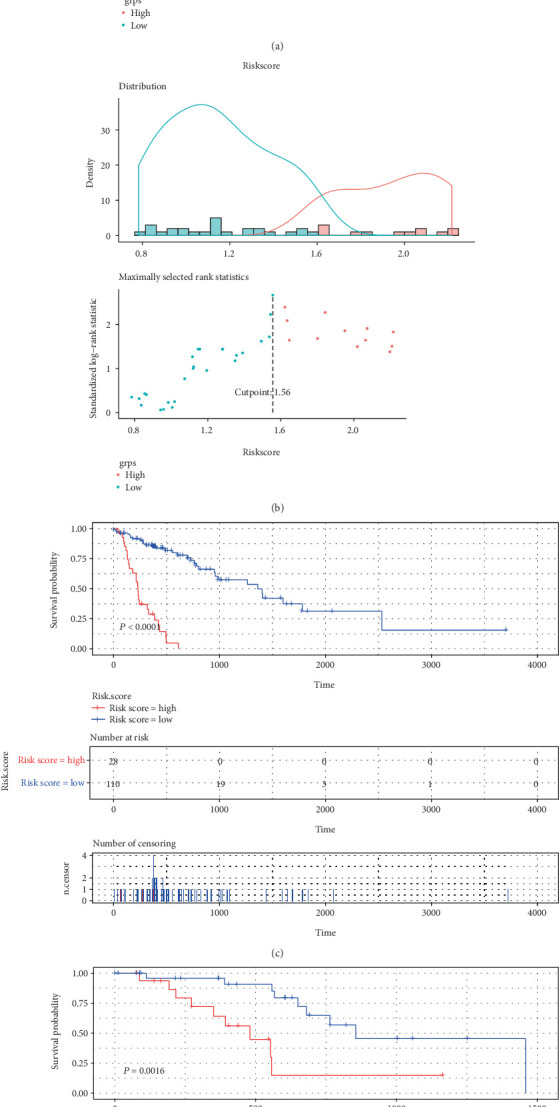
The threshold determination and survival test of the risk score of the prognostic model. The threshold of the cutoff point training set (a) and validation set (b). Survival results of the risk score obtained in the training set (c) and validation set (d) of the prognostic model composed of 4 miRNAs.

Multivariate Cox analysis was carried out for the following 4 DEMs, hsa-miR-1248, hsa-miR-1291, hsa-miR-421, and hsa-miR-7-5p, in the training set and validation set, respectively, and the regression coefficients were obtained (Table 3). Furthermore, the corresponding risk score was calculated for survival analysis and survival test. The threshold determination of the prognostic model was performed. The threshold of the cutoff point in the training set is 1.48 (Figure 6(a)) and in the validation set is 1.56 (Figure 6(b)), respectively. As illustrated in Figures 6(c) and 6(d), the survival analysis results of the risk score obtained by the prognostic model composed of the 4 miRNAs were appropriate in both the training and validation sets (both, *P* < 0.01).

## 4. Discussion

During the past few decades, an increasing number of studies have investigated the causes and potential mechanisms of ESCC tumorigenesis. However, the high incidence and mortality of ESCC worldwide still pose a challenge, as most studies only focus on a single genetic event [9, 26]. Gene profiling or microarray technologies have been widely used to predict potential targets for the treatment of different tumors. In our study, we downloaded three original microarray data and identified 2575, 2111, and 1205 DEGs, as well as 226 DEMs, between ESCC and normal tissues. Moreover, the meta-analysis was used to further obtain more reliable DEGs, and 1001 genes that could be used for sample clustering of each dataset were identified. Furthermore, based on the clinical, survival, and miRNA-seq data downloaded from TCGA, a total of 85 DEMs were found to be significantly associated with the outcome of ESCC patients. As such, a prognostic survival model composed of 4 DEMs, including hsa-miR-1248, hsa-miR-1291, hsa-miR-421, and hsa-miR-7-5p was constructed.

It has been known that tumorigenesis is a complex process that involves the interaction of various genes and signaling pathways [26, 27]. In ESCC, an increasing number of signaling pathways have been reported to play important roles in the progression of the disease [28–30]. Therefore, the analysis of pathways related to neoplasia could provide information regarding tumor development. In the present study, the KEGG pathway analysis revealed that both upregulated and downregulated DEGs were mainly enriched in 18 pathways, such as the DNA replication, cell cycle, p53, proteasome, BcGMP-PKG signaling pathway, valine, leucine, and isoleucine degradation, beta-alanine metabolism, and arginine and proline metabolism signaling pathways. Among these pathways, recent studies have shown a correlation between the proteasome pathway and ESCC carcinogenesis [31]. Zhou et al. [32] revealed that the cell growth and apoptosis of ESCC could be regulated via activation of the p53 signaling pathway. Moreover, the beta-alanine metabolism pathway was reported as a novel subpathway related to ESCC, by the cooperative use of different genes in different pathways [33]. Taken together, the detection of molecules related to these pathways may help predict the occurrence and progression of ESCC.

Furthermore, the PPI network of proteins encoded by the identified DEGs was constructed. It contained 448 nodes and 1144 interactions. *MYC*, with the highest random walk score, could be regarded as the key gene in the PPI network. Indeed, accumulating evidence has demonstrated that *MYC* is an important factor in biological development and tumorigenesis. For instance, Kwon et al. [34] showed that *MYC* was overexpressed in ESCC patients and that its expression could predict better overall survival (OS) for patients. Likewise, Zhong et al. [35] revealed that *MYC* is involved in the tumorigenicity of ESCC by regulating the expression of the hydroxymethylglutaryl coenzyme A reductase (*HMGCR*). Therefore, further research on *MYC* will provide a basis for targeted therapy against ESCC.

miRNAs, which are endogenous, noncoding single-stranded RNAs, have been reported to play critical roles in various biological processes via binding to their target mRNAs [36, 37]. In the present study, a miRNA-target gene regulatory network using the identified DEMs was constructed, and seven miRNAs associated with ESCC, including miR-196a, miR-21, miR-205, miR-194, miR-103, miR-223, and miR-375 were identified in both the microarray data and the miRNA-target gene regulatory network. Among these miRNAs, miR-196a, miR-21, and miR-205 were previously found to be abnormally expressed in ESCC [38–40]. Notably, miR-125a-3p, miR-940, and miR-375 collectively regulated the expression of *MYC*, suggesting that these miRNAs might play a role in ESCC by regulating the *MYC* expression. Additionally, the miRNAs that regulated the same target gene were identified using the coregulatory network, and miR-198a, miR-103, miR-223, miR-21, miR-194, and miR-375 were reported to be miRNAs related to ESCC [41–44]. Survival analysis showed that a total of 85 DEMs were related to prognosis, among which hsa-miR-1248, hsa-miR-1291, hsa-miR-421, and hsa-miR-7-5p were used in a prognostic survival model. Indeed, miR-1248 has been reported to be involved in the microRNA signature model for the prediction of prognosis in patients with the Wilms tumors [45]. Moreover, miR-1291 and miR-421 are associated with OS in patients with lung adenocarcinoma [46, 47]. However, a prognostic survival model using these miRNAs has not been constructed for ESCC. The results of our study revealed that the tumorigenesis of ESCC may be the result of the coregulation of multiple miRNAs. Moreover, several miRNAs, such as hsa-miR-1248, hsa-miR-1291, hsa-miR-421, and hsa-miR-7-5p could be used to construct a prognostic survival model for the prediction of ESCC patient outcomes.

In conclusion, in the present study, we conducted a comprehensive bioinformatics analysis of DEGs, DEMs, and pathways, based on different datasets. As such, we identified DEGs such as *MYC*, miRNAs such as miR-125a-3p, miR-940, miR-375 miR-196a, miR-21, miR-205, miR-194, miR-103, miR-223, and miR-198a, and pathways such as the proteasome, p53, and beta-alanine metabolism pathways, which may be involved in ESCC development. Notably, survival analysis showed that 85 DEMs were related to prognosis, among which hsa-miR-1248, hsa-miR-1291, hsa-miR-421, and hsa-miR-7-5p were used to construct a prognostic survival model. Taken together, these findings have important clinical significance, as they can improve our understanding of the pathogenesis and molecular mechanisms of ESCC. Moreover, our results provide potential biomarkers for the prediction of ESCC prognosis. However, further studies are still needed to confirm the function of the identified genes.

## Figures and Tables

**Table 1:** Signaling pathway enrichment analysis of DEGs in ESCC.

	Term	Count	*P* value
Up	hsa03030:DNA replication	13	3.51*E*-08
	hsa04110:cell cycle	21	8.02*E*-07
	hsa03050:proteasome	11	2.33*E*-05
	hsa03410:base excision repair	9	9.52*E*-05
	hsa03040:spliceosome	17	3.83*E*-04
	hsa03430:mismatch repair	7	4.78*E*-04
	hsa00240:pyrimidine metabolism	14	9.29*E*-04
	hsa03420:nucleotide excision repair	8	5.35*E*-03
	hsa05222:small-cell lung cancer	11	5.50*E*-03
	hsa00480:glutathione metabolism	8	8.42*E*-03
	hsa05230:central carbon metabolism in cancer	9	8.77*E*-03
	hsa04062:chemokine signaling pathway	17	1.18*E*-02
	hsa03013:RNA transport	16	1.29*E*-02
	hsa04510:focal adhesion	17	2.83*E*-02
	hsa00230:purine metabolism	15	3.25*E*-02
	hsa04115:p53 signaling pathway	8	3.39*E*-02
	hsa04512:ECM-receptor interaction	9	4.68*E*-02
	hsa04145:phagosome	13	5.00*E*-02

Down	hsa04810:regulation of actin cytoskeleton	11	4.23*E*-03
	hsa00260:glycine, serine, and threonine metabolism	5	4.86*E*-03
	hsa04022:cGMP-PKG signaling pathway	9	9.30*E*-03
	hsa00280:valine, leucine and isoleucine degradation	5	9.45*E*-03
	hsa00330:arginine and proline metabolism	5	1.17*E*-02
	hsa00072:synthesis and degradation of ketone bodies	3	1.29*E*-02
	hsa00410:beta-alanine metabolism	4	1.72*E*-02
	hsa04710:circadian rhythm	4	1.72*E*-02
	hsa04270:vascular smooth muscle contraction	7	1.91*E*-02
	hsa04924:renin secretion	5	2.68*E*-02
	hsa04713:circadian entrainment	6	2.68*E*-02
	hsa04510:focal adhesion	9	3.02*E*-02
	hsa00982:drug metabolism-cytochrome P450	5	3.25*E*-02
	hsa04610:complement and coagulation cascades	5	3.40*E*-02
	hsa00360:phenylalanine metabolism	3	3.59*E*-02
	hsa04020:calcium signaling pathway	8	4.01*E*-02
	hsa05146:amoebiasis	6	4.02*E*-02
	hsa00071:fatty acid degradation	4	4.30*E*-02

**Table 2:** The key genes (top 20) in the PPI network in ESCC.

Node	Direction	Random walk score
MYC	Up	0.00656632
PCNA	Up	0.005105377
AURKB	Up	0.005066962
STAT1	Up	0.004433487
CXCL12	Down	0.003986094
POLR2K	Up	0.003855735
CDC20	Up	0.003688161
PIK3CA	Up	0.003613145
CDC6	Up	0.003570703
PRKDC	Up	0.00355442
SHMT2	Up	0.003539976
CHEK1	Up	0.003533405
PPARGC1A	Down	0.003496619
MAGOH	Up	0.003355163
SERPINE1	Up	0.003290157
CCR1	Up	0.003277042
LYN	Up	0.003267606
HK2	Up	0.003262363
WDR12	Up	0.003251239
PIK3R1	Down	0.003241252

**Table 3:** The regression coefficients of 4 miRNAs.

Training set		Validation set	
hsa-miR-1248	0.62396	PANTR1	0.37498
hsa-miR-1291	0.40231	LINC01266	0.09693
hsa-miR-421	-0.02532	FGF13-AS1	0.10180
hsa-miR-7-5p	0.19866	TMEM132D-AS1	0.34272

## Data Availability

The data used to support the findings of this study are included within the article.

## Supplementary Materials

Supplementary Materials Supplementary Table 1: 85 miRNAs significantly correlated with prognosis.
