# Supplementary material for: Identification of Differentially Expressed Genes and miRNAs Associated with Esophageal Squamous Cell Carcinoma by Integrated Analysis of Microarray Data
Source: Biomed Res Int. 2020 Jul 1;2020:1980921. doi: 10.1155/2020/1980921 (PMC7352135; doi:10.1155/2020/1980921)
Supplement: Supplementary Materials — Supplementary Table 1: 85 miRNAs significantly correlated with prognosis. [file 1980921.f1.pdf]

Supplementary table1 85 miRNAs significantly correlated with prognosis

miRNA	P value	miRNA	P value
hsa-miR-199a-5p	0.0220	hsa-miR-181a-2-3p	0.0061
hsa-miR-361-3p	0.0373	hsa-miR-181b-5p	0.0046
hsa-miR-362-5p	0.0045	hsa-miR-181c-3p	0.0398
hsa-miR-330-5p	0.0247	hsa-miR-187-3p	0.0172
hsa-miR-331-3p	0.0288	hsa-miR-214-5p	0.0480
hsa-miR-324-5p	0.0085	hsa-miR-214-3p	0.0447
hsa-miR-324-3p	0.0387	hsa-miR-223-3p	0.0020
hsa-miR-339-5p	0.0413	hsa-let-7i-5p	0.0448
hsa-miR-423-5p	0.0124	hsa-miR-128-3p	0.0165
hsa-miR-484	0.0153	hsa-miR-132-5p	0.0011
hsa-miR-491-5p	0.0426	hsa-miR-132-3p	0.0123
hsa-miR-146b-5p	0.0067	hsa-miR-9-5p	0.0086
hsa-miR-146b-3p	0.0320	hsa-miR-9-3p	0.0003
hsa-miR-512-3p	0.0088	hsa-miR-154-3p	0.0237
hsa-miR-501-5p	0.0288	hsa-miR-185-3p	0.0072
hsa-miR-502-5p	0.0452	hsa-miR-194-3p	0.0260
hsa-miR-615-3p	0.0371	hsa-miR-106b-3p	0.0291
hsa-miR-421	0.0050	hsa-miR-200a-5p	0.0138
hsa-miR-767-5p	0.0030	hsa-miR-130b-5p	0.0233
hsa-miR-769-3p	0.0206	hsa-miR-130b-3p	0.0412
hsa-miR-450b-5p	0.0114	hsa-miR-345-5p	0.0163
hsa-miR-940	0.0414	hsa-miR-196b-5p	0.0133
hsa-miR-1291	0.0000	hsa-miR-425-5p	0.0002
hsa-miR-1248	0.0000	hsa-miR-425-3p	0.0151
hsa-miR-1270	0.0180	hsa-miR-431-3p	0.0145

hsa-let-7f-5p	0.0442	hsa-miR-487a-3p	0.0340
hsa-miR-17-5p	0.0060	hsa-miR-503-5p	0.0093
hsa-miR-18a-5p	0.0065	hsa-miR-505-5p	0.0014
hsa-miR-18a-3p	0.0388	hsa-miR-505-3p	0.0047

hsa-miR-21-5p	0.0096	hsa-miR-92b-5p	0.0344
hsa-miR-21-3p	0.0204	hsa-miR-629-5p	0.0111
hsa-miR-22-3p	0.0282	hsa-miR-629-3p	0.0294
hsa-miR-25-3p	0.0229	hsa-miR-652-3p	0.0290
hsa-miR-27a-3p	0.0085	hsa-miR-1301-3p	0.0015
hsa-miR-92a-1-5p	0.0188	hsa-miR-675-3p	0.0274
hsa-miR-93-5p	0.0370	hsa-miR-939-5p	0.0353
hsa-miR-93-3p	0.0179	hsa-miR-1180-3p	0.0266
hsa-miR-105-5p	0.0024	hsa-miR-1226-3p	0.0073
hsa-miR-196a-5p	0.0365	hsa-miR-1287-5p	0.0393
hsa-miR-197-3p	0.0085	hsa-miR-1292-5p	0.0020
hsa-miR-30c-5p	0.0078	hsa-miR-1307-3p	0.0003
hsa-miR-7-5p	0.0004	hsa-miR-3200-3p	0.0194
hsa-miR-181a-5p	0.0273		
